# Preparation and Evaluation of Dental Resin with Antibacterial and Radio-Opaque Functions

**DOI:** 10.3390/ijms14035445

**Published:** 2013-03-07

**Authors:** Jingwei He, Eva Söderling, Pekka K. Vallittu, Lippo V. J. Lassila

**Affiliations:** 1 Department of Biomaterials Science, Institute of Dentistry and BioCity Turku Biomaterial Research Program, University of Turku, Turku 20520, Finland; E-Mails: pekval@utu.fi (P.K.V.); liplas@utu.fi (L.V.J.L.); 2 Turku Clinical Biomaterials Centre-TCBC, University of Turku, Turku 20520, Finland; 3 College of Materials Science and Engineering, South China University of Technology, Guangzhou 510641, China; 4 Institute of Dentistry, University of Turku, Turku 20520, Finland; E-Mail: esoder@utu.fi

**Keywords:** dental resin, radio-opacity, antibacterial activity, mechanical properties, water sorption, water solubility

## Abstract

In order to prepare antibacterial and radio-opaque dental resin, a methacrylate monomer named 2-Dimethyl-2-dodecyl-1-methacryloxyethyl ammonium iodine (DDMAI) with both antibacterial and radio-opaque activities was added into a 2,2-bis[4-(2-hydroxy-3-methacryloyloxypropyl)-phenyl]propane (Bis-GMA)/methyl methacrylate (MMA) dental resin system. Degree of conversion (DC), flexural strength (FS) and modulus (FM), water sorption (WS) and solubility (WSL), antibacterial activity, and radio-opacity (ROX) of the obtained dental resin system were investigated. Bis-GMA/MMA resin system without DDMAI was used as a control. The results showed that DDMAI could endow BIS-GMA/MMA resin system with good antibacterial (*p* < 0.05) and radio-opaque function without influencing the DC (*p* > 0.05). However, incorporating DDMAI into Bis-GMA/MMA resin could reduce mechanical properties (*p* < 0.05) and increase WS and WSL (*p* < 0.05), thus further work is needed in order to optimize the resin formulation.

## 1. Introduction

Since the 1990s, there has been a growing interest in improving properties of dental materials used in restorative dentistry, and many of them are concerned with improving mechanical properties [1–4], decreasing water sorption and solubility [5–8], reducing polymerization shrinkage [7–11], and increasing monomer conversion [12–14]. In addition to these, dental restorative materials also require some other properties, such as antibacterial activity and radio-opacity.

When it was recognized that dental caries formation can be classified as an infectious disease, introduced by cariogenic bacteria, attempts to create restorative materials with antibacterial activity became an attractive topic in dental materials science [15]. A simple way to endow dental restorative materials with antibacterial activity is by incorporating an antibacterial agent into the materials [16–19]. These additives can be released from the material in an aqueous environment to eradicate harmful bacteria. The materials have been tested so as to be incorporated were, e.g., nanosized silver particles and chlorhexidine [20]. However, even a small amount of an antibacterial agent like chlorhexidine can reduce the mechanical properties of dental restorative materials [21], and the release of the agent may exert toxic effects [22]. Furthermore, release of antibacterial agents is based on diffusion and therefore quantities of released substances lower over time, which leads to a limited period of antibacterial effectiveness [23,24].

In order to achieve long-term antibacterial effectiveness, Imazato and his coworkers introduced a concept of the “immobilized bactericide” into dentistry. According to their innovative idea, a quaternary ammonium-containing polymerizable antibacterial monomer methacryloyloxydodecyl-pridinium bromide (MDPB) was synthesized and used in dental restorative materials as antibacterial agents [25]. Studies showed that MDPB containing dental restorative materials had the advantages of long-lasting antibacterial activity and uncompromised mechanical strength under appropriate concentrations [25–28], when compared with agent-releasing antibacterial materials. Subsequently, several polymerizable antibacterial monomers were prepared to formulate long-lasting antibacterial dental restorative materials [29–34].

In general, a polymerizable antibacterial agent always consists of methacrylate group and quaternary ammonium salt structure. The former can immobilize the agent into the polymeric network, and the latter has the main function of killing bacteria. Quaternary ammonium salts are salts of quaternary ammonium cation with an anion, and the anions are usually halogen anions like fluorine anion (F^−^), chlorine anion (Cl^−^), bromine anion (Br^−^), and iodine anion (I^−^). In the halogen elements, iodine is quite radio-opaque because of its high electronic density [35–38]. Therefore, quaternary ammonium methacrylate monomer (QAM) with iodine anion does not only endow methacrylate-based dental resin with antibacterial activity, but also with radio-opacity.

**Figure 1:**
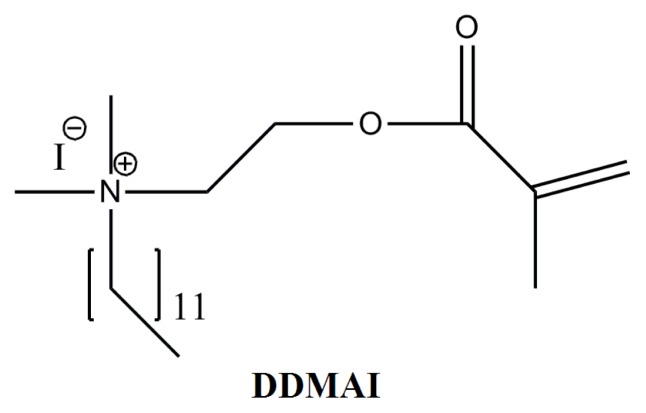
Structure of DDMAI.

In a previous study, a QAM with iodine anion named 2-Dimethyl-2-dodecyl-1-methacryloxyethyl ammonium iodine (DDMAI) (structure was shown in Figure 1) was synthesized [32] and incorporated into the 2,2-Bis[4-(2-hydroxy-3-methacryloyloxypropyl)-phenyl]propane (Bis-GMA)/*tri*-ethyleneglycol dimethacrylate (TEGDMA) (50/50, *wt*/*wt*) dental resin system as an antibacterial and radio-opaque agent [39]. The results showed that polymer with DDMAI had better antibacterial activity and radio-opacity than that of a polymer without DDMAI. However, the maximum mass concentration of DDMAI could not go over 5 wt% due to its miscibility problem with TEGDMA, which led to the situation whereby radio-opacity of DDMAI-containing polymer was not high enough and the polymer only had an inhibition effect on early *Streptococcus mutans* biofilm formation.

As a result of pilot scale research, DDMAI could be mixed well with Bis-GMA even at high concentration. Therefore, higher Bis-GMA concentration is needed for a resin if there is a need to incorporate more DDMAI. In this work, DDMAI was added to a high Bis-GMA concentration containing the resin system Bis-GMA/ methyl methacrylate (MMA) (80/20, *wt*/*wt*) with a series of mass ratios. Antibacterial and radio-opaque activities of obtained polymers were measured to see whether a higher concentration of DDMAI could encourage greater antibacterial activity and radio-opacity. The degree of monomer conversion, water sorption and solubility, and mechanical properties were also examined, in addition to an evaluation of the DDMAI-containing polymers.

## 2. Results

**Figure 2:**
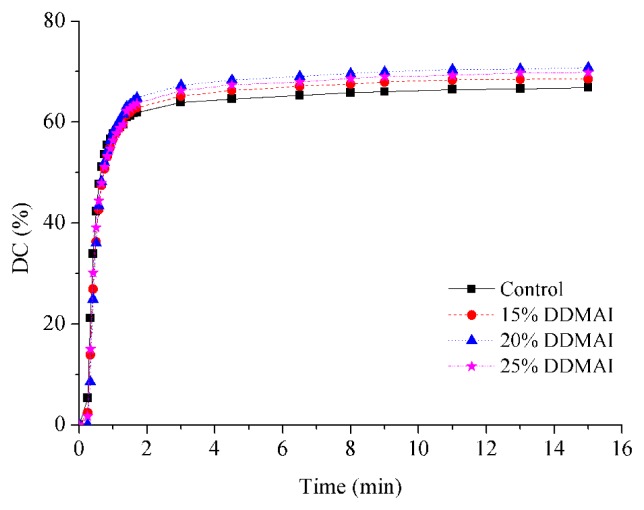
Double-bond conversion of resins with and without DDMAI.

Curves for the change of DC *versus* light polymerization and *post* curing time showed that all of the experimental resins generally had similar polymerization behavior, and the final DC of DDMAI-containing polymers were higher than that of the control polymer (*p* < 0.05) (Figure 2, Table 1).

**Figure 3:**
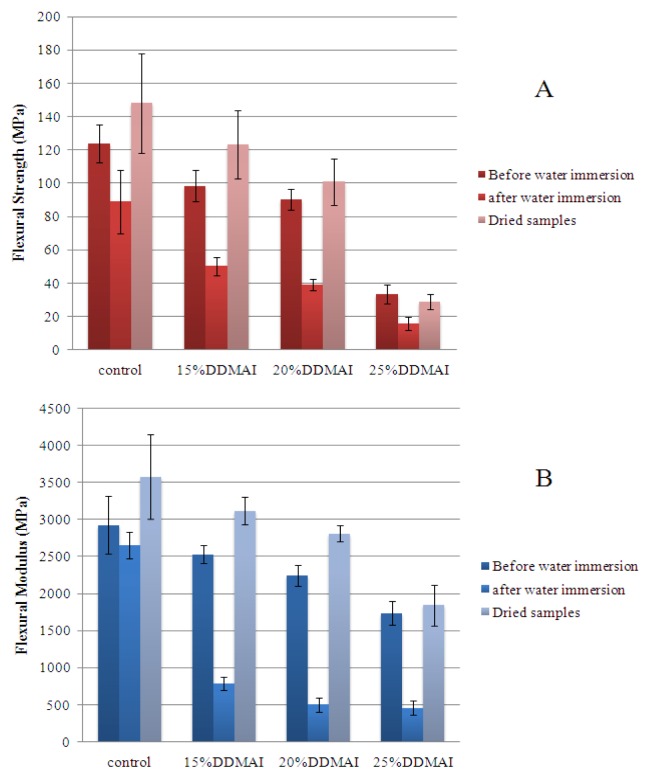
Flexural strength (**A**) and flexural modulus (**B**) of polymers with and without DDMAI before and after water immersion, and after dehydration.

The results of FS and FM were shown in Figure 3. After water immersion, FS and FM decreased significantly (*p* < 0.05), except for the FM in the specimens of control group, which showed a slight decrease of value, and the variation had no statistical meaning (*p* > 0.05). When the water-immersed polymers were dehydrated again, their FS and FM were even higher than the polymers without immersion (*p* < 0.05), except for the FS and FM in the specimens of polymer with 25% DDMAI, which showed no significant differences (*p* > 0.05). Before water immersion, the control polymer had higher FS and FM than all polymers with DDMAI (*p* < 0.05), polymers with 15% and 20% DDMAI had comparable FS and FM (*p* > 0.05), and the polymer with 25% DDMAI had the lowest FS and FM (*p* < 0.05). After water immersion, the control polymer still had the highest FS and FM (*p* < 0.05), polymers with 15% and 20% DDMAI had comparable FS (*p* > 0.05), which was higher than FS of the polymer with 25% DDMAI (*p* < 0.05), and polymers with 20% and 25% DDMAI had comparable FM (*p* > 0.05), which was lower than FM of the polymer with 15% (*p* < 0.05). After dehydration, the polymer with 25% DDMAI had the lowest FS and FM (*p* < 0.05), FS and FM of the control polymer were higher than that of the polymer with 20% DDMAI, and nearly the same as that of polymer with 15% DDMAI (*p* > 0.05). There were no significant differences of FS and FM between polymers with 15% and 20% DDMAI (*p* > 0.05). In general, no matter whether the samples were taken before water immersion, after water immersion, or once dried again, the control polymer had the highest FS and FM, and the trend of FS and FM of polymers with DDMAI was that FS and FM decreased with the increase of DDMAI concentration.

The values of water sorption (WS) and solubility (WSL) are listed in Table 1. All of DDMAI-containing polymers had higher WS and WSL than the control polymer (*p* < 0.05). The polymer with 25% DDMAI had the highest WS and WSL (*p* < 0.05), and polymers with 15% and 20% DDMAI had comparable WS and WSL (*p* > 0.05).

**Figure 4:**
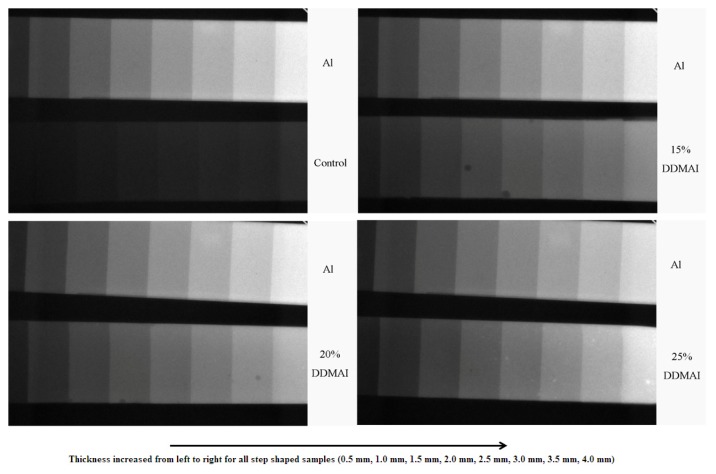
X-ray graph of polymers (thickness from 0.5 to 4 mm) with and without DDMAI, and an aluminum steps (thickness from 0.5 to 4 mm) was used as reference. Thickness increased from left to right.

The radiograph revealed that radio-opacity increased with the increasing of the thickness of the polymer and the amount of DDMAI in the polymer (Figure 4). Using the aluminum as the radio-opacity standard, ROX of polymer with DDMAI was significantly higher than that of the control polymer, but was still lower than that of aluminum (Table 1).

**Figure 5:**
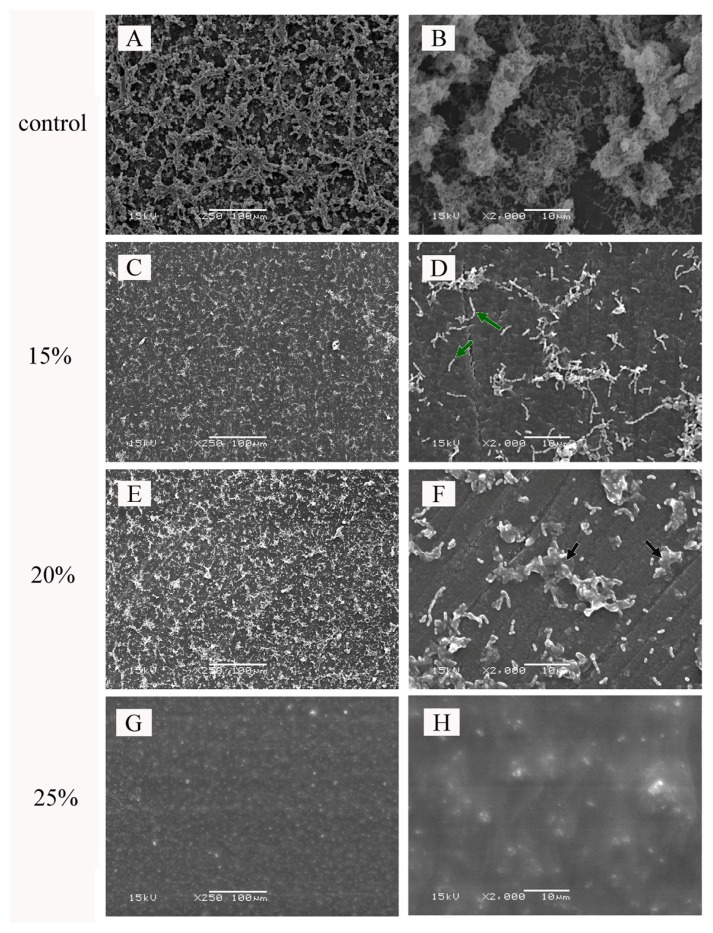
SEM micrographs of biofilm inhibition test specimens before water immersion: control polymer (**A**,**B**), polymer containing 15 wt% (**C**,**D**), 20 wt% (**E**,**F**), and 25 wt% (**G**,**H**) DDMAI. (magnification).

The amounts of viable bacteria in the biofilm formed on the surface of polymer with and without DDMAI were shown in Table 1. Before water immersion, the amount of bacteria on the surface of control polymer was much higher than that found on the polymers with DDMAI (*p* < 0.05), and on the surface of some DDMAI-containing polymers, no viable bacteria were detected. The SEM micrographs of polymers with and without DDMAI for biofilm inhibition tests before water immersion were shown in Figure 5. A thick biofilm was formed on the surface of the control polymer (Figure 5A,B). However, on the surface of the polymers with DDMAI, a concentration-dependent inhibition of biofilm formation was observed. For the polymers with 15% and 20% DDMAI, biofilm formation was strongly reduced (Figure 5C–F). In lower magnification micrographs (×250), the polymer with 20% DDMAI (Figure 5E) seemed to accumulate more bacteria than the polymer with 15% DDMAI (Figure 5C), but in higher magnification micrographs (×2000), it could be seen clearly that most bacteria on the surface of the polymer with 20% DDMAI (Figure 5F) were dead (as pointed out by the black arrow), whereas most bacteria on the surface of the polymer with 15% DDMAI (Figure 5D) were still alive (as shown by the green arrow). For the polymer with 25% DDMAI, no biofilm was detected (Figure 5G,H). After water immersion, the number of bacteria formed on the surface of the polymer with DDMAI was more than that of the DDMAI-containing polymer without water immersion (as shown in Table 1), but still significantly less than that of the control polymer (*p* < 0.05).

## 3. Discussion

Quaternary ammonium compounds are well known as effective antibacterial agents, and have already been used in many fields, such as water treatment, food applications, textile products, health care products, and wound-healing [40–44]. DDMAI used in this study is a quaternary ammonium compound synthesized by reacting DMAEMA with 1-Iodododecane through Menschutkin reaction [32]. Studies revealed that DDMAI had strong antibacterial activity against *S. mutans*[32], and the polymer with 5 wt% DDMAI exhibited inhibitory effect on young *S. mutans* biofilm formation [39]. In addition, the polymer with DDMAI also presented radio-opaque activity [39], which is an important property for all dental materials. However, like some monomethacrylates with pendant quaternary ammonium moieties, DDMAI also showed miscibility problem with hydrophobic dimethacrylates used in dental composites [33], and the polymer with low concentration of DDMAI could not show obvious antibacterial and radio-opaque activity. In order to increase the DDMAI concentration, resin should be designed to have solubility parameter similar to DDMAI. We found that DDMAI could be miscible with Bis-GMA, which is an important dimethacrylates to dental materials, so DDMAI concentration could be increased if the amount of Bis-GMA in resin formulation was increased. Therefore, a resin system (Bis-GMA/MMA, 80/20, *wt*/*wt*) with high Bis-GMA concentration was prepared, and DDMAI was added into this resin system with a series of the mass ratios as 15%, 20%, and 25%.

From the results of double-bond conversion, it could be seen that incorporation of DDMAI could increase the DC of resin. It is well known that DC is governed largely by the vitrification point, and DC decreased with the increasing of concentration of Bis-GMA in the resin system [33,45]. The higher DC of DDMAI-containing polymers is likely due to an overall reduction in the amount of Bis-GMA when DDMAI was added. Moreover, cross-linking density of a polymer should be decreased when a large amount of DDMAI is incorporated, for there is only one methacrylate in the structure of DDMAI, and this could make the polymer chain more flexible, prolong the vitrification time, and lead to higher DC [46].

It has already been reported that the advantage of quaternary ammonium methacrylates is they can endow methacrylate resin based dental materials with antibacterial activity without undermining the mechanical properties [26,39,47], but it is limited to an appropriate concentration. When the concentration of quaternary ammonium methacrylates is beyond a certain limit, mechanical strength could be decreased significantly [34,48,49]. This phenomenon was also observed in this work. All of the polymers with DDMAI had lower FS and FM than that of the control polymer, and FS and FM decreased with the increasing of DDMAI concentration (Figure 3). This means that 15 wt% of DDMAI has already exceeded the concentration limit of DDMAI in Bis-GMA/MMA resin system. The lower FS and FM of DDMAI-containing polymers is mainly attributed to the mono-structure of DDMAI and the decrease in the amount of Bisphenol A structure, because the former can decrease the cross-linking density of the polymer, the latter can decrease the enhancement structure of the polymer, and both of these two factors can decrease FS and FM of the relevant polymer. Water absorbed by the polymer can serve as plasticizer in the polymer and reduce the mechanical properties of it [49–51], so nearly all of experimental polymers had lower FS and FM after water immersion here (Figure 3). From Figure 3, it could be seen that reduction rates of FS and FM of DDMAI-containing polymers were higher than that of the control polymer; this is likely due to the higher water sorption of DDMAI-containing polymers (as shown in Table 1). In the structure of DDMAI, there exist both positive and negative charges, which can absorb water [48,52], so water sorption of DDMAI-containing polymers were higher than that of the control polymer. Compared with control polymer, DDMAI-containing polymers had higher water solubility (Table 1), which means there were more unreacted monomers left in the network of DDMAI-containing polymers. Unreacted monomers could also be monomers of MMA, which should be taken into account in interpretation of results. Photoinitiated polymerization of MMA may occur but it requires irradiation time of a couple of hours before adequate degree of monomer conversion is reached [53,54]. A change in flexural properties after dehydration were found to be higher than before sorption, thus reflecting that there were residuals in the polymer which have plasticized the matrix. After being stored in water, residuals such as the unreacted monomers leached out from the polymer, and the polymer of higher strength and modulus of elasticity was obtained as described earlier [55].

Figure 4 reveals that incorporating DDMAI could increase radio-opacity of a dental polymer, and radio-opacity increased with the increasing of thickness of the polymer and the amount of DDMAI in the polymer. Though DDMAI-containing polymers in this research have much better radio-opacity than that of control polymer, they are still not adequate to be used in clinical applications directly. According to ISO 4049:1988 (E), resin-based materials used in dentistry should have greater radio-opacity than that of the same thickness of aluminum, and the radio-opacity of DDMAI-containing polymers here are all less than that of the same thickness of aluminum (Table 1). However, this kind of dental resin might have potential to be used in fiber-reinforced composites which lack radio-opacity, and where radio-opaque fillers could not be added in.

Even though the present ecological plaque hypothesis emphasizes that non-*mutans* bacteria may be the key microorganisms responsible for maintaining dynamic stability on tooth surface, *S. mutans* has a central role in the initiation of dental caries on enamel and root surface [56]. Therefore, a single-species biofilm model with *S. mutans* as the testing organism was used to evaluate the antibacterial property of the DDMAI-containing polymers. For the materials which were not immersed in water, the results of the viability tests (Table 1) and SEM experiments (Figure 5) were in good agreement. Both 15% and 20% DDMAI strongly inhibited biofilm formation and for 25% DDMAI no viable cells or visible biofilm could be detected on the material surfaces. This means that the antimicrobial effect of DDMAI was so strong that it inhibited both adhesion and growth of *S. mutans*.

Two different mechanisms could be used to explain the biofilm inhibitory effect observed in this study on the DDMAI-containing polymers: (I) the release of unreacted DDMAI from the polymer, which can devitalize planktonic *S. mutans*, thus reducing the number of bacteria available for adherence [57–59]; and (II) DDMAI immobilized in the polymer network, killing the bacteria which attach to the surface of the polymer. This can inhibit bacterial growth and biofilm formation on the polymer surface [57,60,61]. Based on the present data, mechanism (I) might be the dominating reason for biofilm inhibition of polymer containing 25% DDMAI before water immersion, because no bacteria could be seen (Figure 5G,H) or recovered (Table 1) from its surface. After water immersion, the effecy of mechanism (I) should be decreased because of the decrease in the amount of leachable DDMAI (water solubility mechanism of DDMAI should be investigated in the future to confirm whether there still remains leachable DDMAI or not after 35 days of water immersion), and the immobilized DDMAI might become the main reason for biofilm inhibition. However, antibacterial activity of QAM would be reduced after immobilization [27], so that is why more bacteria were recovered from the surface of DDMAI-containing polymers after water immersion.

Though DDMAI-containing polymers had lower FS and FM than that of the control polymer, FS (98.4 MPa before water immersion, 50.3 MPa after water immersion, and 123.4 MPa after dehydration) of the polymer with 15% DDMAI met the requirement for dental resin-based restorative materials in respect to FS (ISO 4049: 1988 (E)), which should be not lower than 50 MPa. Therefore, the polymer with 15% DDMAI still had the potential to be used as dental restorative material in light of its effective antibacterial activity.

## 4. Experimental Section

### 4.1. Materials

2-Dimethyl-2-dodecyl-1-methacryloxyethyl ammonium iodine (DDMAI) was synthesized according to the literature [32]. 2,2-Bis[4-(2-hydroxy-3-methacryloyloxypropyl)-phenyl]propane (Bis-GMA) was purchased from Esstech Inc. (Essington, PA, USA), methyl methacrylate (MMA), camphoroquinone (CQ), and *N*,*N*′-dimethyl aminoethyl methacrylate (DMAEMA) were purchased from Sigma-Aldrich Co. (St. Louis, MO, USA). All of the reagents were used without purification.

### 4.2. Methods

DDMAI was added into Bis-GMA/MMA (80/20, wt/wt) resin system with mass ratios of 15 wt%, 20 wt%, and 25 wt%, CQ (0.7 wt%) and DMAEMA (0.7 wt%) were mixed as a photoinitiator system. MMA was used as diluent in this research because of its low viscosity. Though MMA has low reactivity in light polymerization, it is well known for its biological effects and has lower cytotoxicity than TEGDMA [62,63].

Resin without DDMAI was used as a control in all of measurements. All of the compounds were well blended to obtain a homogeneous mixture, and stored at darkness before use.

#### 4.2.1. Monomer (Double-Bond) Conversion

The degree of monomer conversion (DC) was determined by using an FTIR spectrometer (Spectrum One, Perkin-Elmer, Waltham, MA, USA) with an attenuated total reflectance (ATR) accessory. The FTIR spectra were recorded with one scan at a resolution of 4 cm^−1^. All the samples were analyzed in a mold that was 2 mm thick and 6 mm in diameter. Then, the sample was irradiated for 60 s with a visible light-curing unit (Curing Light 2500, *λ* = 400–520 nm, *I* ≈ 550 mW cm^−2^, 3M Co., St Paul, MN, USA). The sample was scanned for its FTIR spectrum every 5 s until 15 min after the beginning of irradiation.

**Figure 6:**
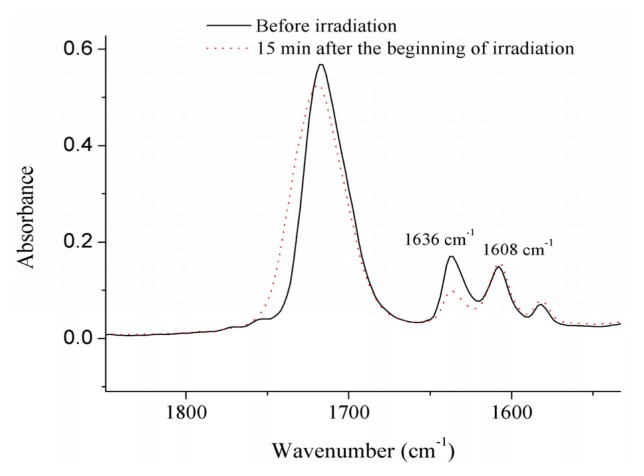
Variation of the absorption peak at 1636 cm^−1^ with respect to the peak at 1608 cm^−1^, before and after irradiation.

To determine the percentage of reacted double bonds, the absorbance intensities of the methacrylate C=C absorbance peak at 1636 cm^−1^, which were decreased after being irradiated (as shown in Figure 6), and an internal phenyl ring standard peak at 1608 cm^−1^, were calculated using a baseline method. The ratios of absorbance intensities were calculated and compared before and after polymerization. The DC at every irradiation time was calculated by using the Equation:

(1) $$\text{DC}(t)=[1-{\left(A_{\text{c}=\text{c}}/A_{\text{ph}}\right)}_{t}/{\left(A_{\text{c}=\text{c}}/A_{\text{ph}}\right)}_{0}]\times 100\%$$

where *A*_C=C_ and *A*_ph_ are the absorbance intensity of methacrylate C=C at 1636 cm^−1^ and phenyl ring at 1608 cm^−1^, respectively; (*A*_C=C_/*A*_ph_)_0_ and (*A*_C=C_/*A*_ph_)*_t_* are the normalized absorbance of functional group at the radiation time 0 and t, respectively; DC(*t*) is the conversion of methacrylate C=C as a functional of radiation time.

#### 4.2.2. Flexural Strength and Modulus

Eight specimens were prepared for every sample formulation (size 2 × 2 × 25 mm^3^). Three-point bending test (span 20 mm) was carried out to evaluate the flexural strength (FS) and modulus (FM) according to ISO 4049:1988 (E) standard with a material testing machine (model LRX, Lloyd Instrument Ltd., Fareham, England), at a cross-head speed of 1.00 mm/min. FS and FM of specimens for every sample formulation, which were immersed in distilled water at 37 °C for 35 days (equivalent to the immersion time of water sorption and solubility tests), were also measured.

#### 4.2.3. Water Sorption and Solubility

Eight specimens for every sample formulation were prepared as the size of specimens for the three-point bending test. The dry weight (M_d_) of every specimen was measured with a scale (Mettler A30, Mettler Instrument Co., Highstone, NJ, USA) with an accuracy of 0.1 mg. Subsequently, the specimens were immersed in 30 mL of distilled water at 37 °C. At fixed time intervals, they were removed, blotted dry to remove excess water, massed and returned to the water. Equilibrium mass (*M*_e_) was obtained until there was no significant change in mass at 35 days immersion. The specimens were then dried for 7 days at 60 °C until their mass was constant, and the result was recorded as *M*_f_. Water sorption (*WS*) and solubility (*WSL*) were then calculated using the following formulae:

(2) $$WS=\frac{M_{e}-M_{f}}{M_{f}}\times 100\%\;WSL=\frac{M_{d}-M_{f}}{M_{d}}\times 100\%$$

#### 4.2.4. Radio-Opacity

Resins were poured into step-shaped mold (thickness from 0.5 to 4 mm, with 0.5 mm steps), then covered with mylar stripe and topped by a glass plate. The specimens were light cured for 60 s on each part until all the portions were irradiated. The cured sample steps were irradiated with X-ray (63 kV, 8 mA, 0.008 s) to get a radiograph. The relative radio-opacity was determined by comparison with the opacity exhibited by a standard aluminum step-wedge (0.5–4 mm) exposed on the same radiograph. A free image editing software ImageJ was used to measure the gray value of the sample and aluminum in the resulting image. The relative radio-opacity (ROX) of every sample was calculated by using the Equation:

(3) $$\text{ROX}=[G_{\text{d}}-G_{\text{b}}]/[G_{\text{a}}-G_{\text{b}}]\times 100\%$$

where *G*_d_, *G*_b_, and *G*_a_ are the gray value of disc, background and aluminum at the same thickness, respectively.

#### 4.2.5. Biofilm Inhibition Test

Six disc-shaped samples (2 mm thick and 8 mm in diameter) for every resin formulation were prepared for biofilm inhibition test; three of them were tested before water immersion, and the other three samples were tested after water immersion. Every disc was polished with 4000 grit (FEPA) grinding paper. Biofilm inhibition test of the discs, which were soaked in distilled water at 37 °C for 35 days (as long as the immersion time in water sorption and solubility test), was also taken to see whether all of samples had long-term inhibition effectiveness. Before the biofilm inhibition test, all of the samples were sterilized by ultraviolet light for 30 min to avoid cross-contamination.

The inhibition of biofilm formation reflecting plaque accumulation was tested by a modification of the method originally presented by Ebi *et al.*[64]. The microorganism we used was the reference strain *Streptoccus mutans* Ingbritt. It was first grown overnight in Brain Heart Infusion medium (BHI; Becton Dickinson and Company, Sparks, MD, USA). After 24 h, the cells were washed with phosphate-buffered saline (5000 × *g*, 10 min) and then they were suspended in BHI containing 1% sucrose (*A*_550_ = 0.05). This suspension (500 μL) was pipetted onto the experimental discs placed in the wells of cell culture plates. The plates were incubated anaerobically (90% N_2_, 5% CO_2_, 5% H_2_) at 37 °C for 24 h.

The biofilms were collected with microbrushes (Quick-Stick®, Dentsolv AB, Saltsjö-Boo, Sweden) from the disc surface exposed to the medium to test tubes containing Tryptic Soy Broth (Becton Dickinson and Company, Franklin Lakes, NJ, USA). The tubes were vortexed and mildly sonicated and then serially diluted for plate culturing of *S. mutans.* The plates were grown for three days anaerobically (80% N_2_, 10% CO_2_, 10% H_2_) at +37 °C on Mitis salivarius agar (Becton Dickinson and Company, Franklin Lakes, NJ, USA), the colonies were counted under a stereomicroscope and results expressed as colony-forming units (CFU)/disc surface. The biofilm collection method has been tested in our earlier studies and it is highly reproducible [65,66].

In some experiments, the materials were subjected to fixation followed by scanning electron microscope (SEM), as described in the following.

#### 4.2.6. Scanning Electron Microscope (SEM)

For the SEM examinations, the samples were fixed for 5 min (2% glutaraldehyde and 2% formaldehyde in phosphate-buffered saline, pH 7.4 (Orion Diagnostica, Espoo, Finland), rinsed once in distilled water, and dried in an ascending ethanol series: 50% ethanol for 5 min, 70% ethanol for 10 min, two times 96% ethanol for 10 min, and absolute ethanol for 5 min. Finally, the specimens were sputter coated with gold and examined with SEM (Model JSM 5500, JEOL Ltd., Tokyo, Japan) at magnifications of 250×, and 2000×.

#### 4.2.7. Statistical Analysis

The results of the double-bond conversion, three-point bending test, and biofilm inhibition test were statistically analyzed with analysis of variance (ANOVA) at the *p* < 0.05 significance level by software SPSS 13.0 (SPSS Inc.: Chicago, CA, USA, 2004). Subsequent multiple comparisons were conducted using Tukey’s *post hoc* analysis.

## 5. Conclusions

DDMAI was added into Bis-GMA/MMA (80/20, *wt*/*wt*) with a series of mass ratios (from 15 to 25 wt%). Though the obtained polymers had higher DC, better radio-opacity, and significant antibacterial activity when compared with the polymer without DDMAI, the lower flexural strength and modulus, higher water sorption and solubility are parameters that need further optimization for preclinical research with the monomer system.

## Figures and Tables

**Table 1:** Double-bond conversion, water sorption and solubility, relative X-ray opacity, and *Streptococcus mutans* colonies in biofilm on the surface of polymers with and without DDMAI.

Resin system	DC (%)	WS (%)	WSL (%)	ROX (%)	*Streptococcus mutans* colonies (Log CFU/mm^2^ × 10^2^)	

					Before water immersion	After water immersion
Control	66.8 ± 1.3 ^a^	3.47 ± 0.20 ^a^	0.60 ± 0.05 ^a^	11.4	13.61 ± 0.39 ^a^	14.94 ± 0.25 ^a^
15% DDMAI	68.5 ± 1.0 ^b^	4.06 ± 0.17 ^b^	1.13 ± 0.11 ^b^	51.2	2.17 ± 4.35 ^b^	12.37 ± 0.43 ^b^
20% DDMAI	70.8 ± 0.7 ^c^	4.37 ± 0.08 ^b^	1.34 ± 0.06 ^b^	67.7	1.62 ± 3.24 ^b^	12.56 ± 0.20 ^b^
25% DDMAI	69.8 ± 1.1 ^b,c^	8.38 ± 0.98 ^c^	3.18 ± 0.64 ^c^	85.9	0.00 ± 0.00 ^b^	10.30 ± 0.21 ^c^

Lower case letters indicate statistical differences within a column (Tukey’s test, *p* = 0.05).

## References

[1] Atai M., Nekoomanesh M., Hashemi S.A., Amani S. (2004). Physical and mechanical properties of an experimental dental composite based on a new monomer. Dent. Mater.

[2] Pereira S.G., Osorio R., Toledano M., Nunes T.G. (2005). Evaluation of two Bis-GMA analogues as potential monomer diluents to improve the mechanical properties of light-cured composite resins. Dent. Mater.

[3] Lee V.A., Cardenas H.L., Rawls H.R. (2010). Rubber-toughening of dimethacrylate dental composite resin. J. Biomed. Mater. Res. B.

[4] Kurata S., Yamazaki N. (2011). Synthesis of dimethacryloxy ethyl-1,1,6,6-tetrahydro-perfluorohexamethylene- 1,6-dicarbamate as dental base monomers and the mechanical properties of the copolymers of the monomer and methy methacrylate. Dent. Mater. J.

[5] Khatri C.A., Stansbury J.W., Schultheisz C.R., Antonucci J.M. (2003). Synthesis, characterization and evaluation of urethane derivatives of Bis-GMA. Dent. Mater.

[6] Sideridou I.D., Achilias D.S., Spyoudi C., Karabela M. (2004). Water sorption characteristics of light-cured dental resins and composites based on Bis-EMA/PCDMA. Biomaterials.

[7] Kim J.G., Chung C.M. (2003). Trifunctional methacrylate monomers and their photocured composites with reduced curing shrinkage, water sorption, and water solubility. Biomaterials.

[8] He J.W., Liu F., Luo Y.F., Jia D.M. (2012). Synthesis and characterization of a dimethacrylates monomer with low shrinkage and water sorption for dental application. J. Appl. Polym. Sci.

[9] He J.W., Liao L.L., Liu F., Luo Y.F., Jia D.M. (2010). Synthesis and characterization of a new dimethacrylate monomer based on 5,5-bis(4-hydroxylphenyl)-hexahydro-4,7-methanoindan for root canal sealer application. J. Mater. Sci. Mater. Med.

[10] He J.W., Luo Y.F., Liu F., Jia D.M. (2010). Synthesis, characterization and photopolymerization of a new dimethacrylate monomer based on (alpha-methyl-benzylidene)bisphenol used as root canal sealer. J. Biomater Sci. Polym. Ed.

[11] He J.W., Luo Y.F., Liu F., Jia D.M. (2010). Synthesis and characterization of a new trimethacrylate monomer with low polymerization shrinkage and its application in dental restoration materials. J. Biomater. Appl.

[12] Viljanen E.K., Skrifvars M., Vallittu P.K. (2004). Degree of conversion of an experimental monomer and methyl methacrylate copolymer for dental applications. J. Appl. Polym. Sci.

[13] Viljanen E.K., Skrifvars M., Vallittu P.K. (2005). Dendrimer/methyl methacrylate copolymers: residual methyl methacrylate and degree of conversion. J. Biomater. Sci. Polym. Ed.

[14] Viljanen E.K., langer S., Skrifvars M., Vallittu P.K. (2006). Analysis of residual monomers by HPLC and HS-GC/MS. Dent. Mater.

[15] Imazato S., Chen J.H., Ma S., Izutani N., Li F. (2012). Antibacterial resin monomers based on quaternary ammonium and their benefits in restorative dentistry. Jpn. Dent. Sci. Rev.

[16] Shay D.E., Allen T.J., Mantz R.F. (1956). The antibacterial effects of some dental restorative materials. J. Dent. Res.

[17] Ehara A., Torii M., Imazato S., Ebisu S. (2000). Antibacterial activities and release kinetics of a newly developed recoverable controlled agent-release system. J. Dent. Res.

[18] Leung D., Spratt D.A., Pratten J., Gulabivala K., Mordan N.J., Young A.M. (2005). Chlorhexidine-releasing methacrylate dental composite materials. Biomaterials.

[19] Al-Musallam T.A., Evans C.A., Drummond J.L., Matasa C., Wu C.D. (2006). Antimicrobial properties of an orthodontic adhesive combined with cetylpyridinium chloride. Am. J. Orthod. Dentofacial. Orthop.

[20] Nganga S., Travan A., Donati I., Crosera M., Paoletti S., Vallittu P.K. (2012). Degradation of silver-polysaccharide nanocomposite in solution and as coating on fiber-reinforced composites by lysozyme and hydrogen peroxide. Biomacromolecules.

[21] Jedrychowski J.R., Caputo A.A., Kerper S. (1983). Antibacterial and mechanical properties of restorative materials combined with chlorhexidines. J. Oral. Rehabil.

[22] Wilson S.J., Wilson H.J. (1993). The release of chlorhexidine from modified dental acrylic resin. J. Oral. Rehabil.

[23] Waltimo T., Luo G., Samaranayake L.P., Vallittu P.K. (2004). Glass fibre-reinforced composite laced with chlorhexidine diglucoonate and yeast adhesion. J. Mater. Sci. Mater. Med.

[24] Lahdenperä M.S., Puska M.A., Alander P.M., Waltimo T., Vallittu P.K. (2004). Release of chlorhexidine digluconate and flexural properties of glass fibre reinforced provisional fixed partial denture polymer. J. Mater. Sci. Mater. Med.

[25] Imazato S., Torii M., Tsuchitani Y. (1993). Immobilization of an antibacterial component in composite resin. Dent. Jpn.

[26] Imazato S., Torii M., Tsuchitani Y., McCabe J.F., Russell R.R.B. (1994). Incorporation of bacterial inhibitor into resin composite. J. Dent. Res.

[27] Imazato S., Russell R.R.B., McCabe J.F. (1995). Antibacterial activity of MDPB polymer incorporated in dental resin. J. Dent.

[28] Imazato S. (2003). Antibacterial properties of resin composites and dentin bonding systems. Dent. Mater.

[29] Xiao Y.H., Ma S., Chen J.H., Chai Z.G., Li F., Wang Y.J. (2009). Antibacterial activity and bonding ability of an adhesive incorporating an antibacterial monomer DMA-CB. J. Biomed. Mater. Res. B.

[30] Huang L., Xiao Y.H., Xing X.D., Li F., Ma S., Qi L.L., Chen J.H. (2011). Antibacterial activity and cytotoxicity of two novel cross-linking antibacterial monomers on oral pathogens. Arch. Oral Biol.

[31] Xie D., Weng Y.M., Guo X., Zhao J., Gregory R.L., Zheng C. (2011). Preparation and evaluation of a novel glass-ionomer cement with antibacterial functions. Dent. Mater.

[32] He J.W., Söderling E., Österblad M., Vallittu P.K., Lassila L.V.J. (2011). Synthesis of methacrylate monomers with antibacterial effects against *S. Mutans*. Molecules.

[33] Antonucci J.M., Zeiger D.N., Tang K., Lin-Gibson S., Fowler B.O., Lin N.J. (2012). Synthesis and characterization of dimethacrylates containing quaternary ammonium functionalities for dental applications. Dent. Mater.

[34] Xu X., Wang Y., Liao S., Wen Z.T., Fan Y. (2012). Synthesis and characterization of antibacterial dental monomers and composites. J. Biomed. Mater. Res. B.

[35] Davy K.W.M., Anseau M.R., Odlyha M., Foster G.M. (1997). X-ray opaque methacrylate polymers for biomedical applications. Polym. Int.

[36] Davy K.W.M., Anseau M.R., Berry C. (1997). Iodinated methacrylate copolymers as X-ray opaque denture base acrylics. J. Dent.

[37] Galperin A., Margel S. (2006). Synthesis and characterization of new micrometer-sized radio-opaque polymeric particles of narrow size distribution by a single-step swelling of uniform polystyrene template microspheres for X-ray imaging applications. Biomacromolecules.

[38] Galperin A., Margel D., Margel S. (2006). Synthesis and characterization of uniform radio-opaque polystyrene microspheres for X-ray imaging by a single-step swelling process. J. Biomed. Mater. Res. A.

[39] He J., Söderling E., Lassila L.V.J., Vallittu P.K. (2012). Incorporation of an antibacterial and radio-opaque monomer into dental resin system. Dent. Mater.

[40] Kanazawa A., Ikeda T., Endo T. (1993). Polymeric phosphonium salts as novel class of cationic biocides. III. Immobilization of phosphonium salts by surface photografting and antibacterial activity of the surface-treated polymer-films. J. Polym. Sci. Pol. Chem.

[41] Worley S.D., Sun G. (1996). Biocidal polymers. Trends Polym. Sci.

[42] Kenawy E.R., Worley S.D., Broughton R. (2007). The chemistry and applications of antimicrobial polymers: A state-of-art review. Biomacromolecules.

[43] Moran J., Addy M., Jackson R., Newcombe R.G. (2000). Comparative effects of quaternary ammonium mouthrinses on 4-day plaque regrowth. J. Clin. Periodontol.

[44] Simoncic B., Tomsic B. (2010). Structure of novel antimicrobial agents for textile—A review. Text. Res. J.

[45] Goncalves F., Kawano Y., Pfeifer C., Stansbury J.W., Braga R.R. (2009). Influence of Bis-GMA, TEGDMA, and BisEMA contents on viscosity, conversion, and flexural strength of experimental resins and composites. Eur. J. Oral. Sci.

[46] Lu H., Stansbury J.W., Nie J., Berchtold K.A., Bowman C.N. (2005). Development of highly reactive mono-(meth)acrylates as reactive diluents for dimethacrylate-based dental resin systems. Biomaterials.

[47] He J., Söderling E., Vallittu P.K., Lassila L.V.J. (2012). Investigation of double bond conversion, mechanical properties, and antibacterial activity of dental resins with different alkyl chain length quaternary ammonium methacrylate monomers (QAM). J. Biomater. Sci. Polym. E.

[48] Weng Y., Guo X., Chong V.J., Howard L., Gregory R.L., Xie D. (2011). Synthesis and evaluation of a novel antibacterial dental resin composite with quaternary ammonium salts. J. Biomed. Sci. Eng.

[49] Weng Y., Howard L., Guo X., Gong Y.J., Gregory R.L., Xie D. (2012). A novel antibacterial resin composite for improved dental restoratives. J. Mater. Sci.Mater. Med.

[50] Dermaut W., van den Kerkhof T., van der Veken B.J., Mertens R., Geise H.J. (2000). Cold stretching of PPV with water as a plasticizer. Macromolecules.

[51] Sideridou I.D., Karabela M.M., Voucoudi E.C. (2008). Dynamic thermomechanical properties and sorption characteristics of two commercial light cured dental resin composites. Dent. Mater.

[52] Wang G., Weng Y., Chu D., Xie D., Chen R. (2009). Preparation of alkaline anion exchange membranes based on functional poly(ether-imide) polymers for potential fuel cell applications. J. Membr. Sci.

[53] Kasapoglu F., Aydin M., Arsu N., Yagci Y. (2003). Photoinitiated polymerization of methyl methacrylate by phenacyl type salts. J. Photochem. Photobiol. A.

[54] Kiskan B., Zhang J., Wang X., Antonietti M., Yagc I.Y. (2012). Mescoporous graphitic carbon nitride as a heterogeneous visible light photoinitiator for radical polymerization. ACS Macro. Lett..

[55] Lassila L.V.J., Nohrström T., Vallittu P.K. (2002). The influence of short-term water storage on the flexural properties of unidirectional glass fiber-reinforced composite. Biomaterials.

[56] Tanzer J.M., Livingston J., Thompson A.M. (2001). The microbiology of primary dental caries in humans. J. Dent. Educ.

[57] Rupf S., Balkenhol M., Sahrhage T.O., Baum A., Chromik J.N., Ruppert K., Wissenbachc D.K., Maurerc H.H., Hanniga M. (2012). Biofilm inhibition by an experimental dental resin composite containing octenidine dihydrochloride. Dent. Mater.

[58] Balamurugan A., Balossier G., Laurent-Maquin D., Pina S., Rebelo A.H., Faure J., Ferreira J.M. (2008). An *in vitro* biological and anti-bacterial study on a sol-gel derived silver-incorporated bioglass system. Dent. Mater.

[59] Türkün L.S., Türkün M., Ertuğrul F., Ateş M., Brugger S. (2008). Long-term antibacterial effect and physical properties of a chlorhexidine-containing glass ionomer cement. J. Esthet. Restor. Dent.

[60] Giammanco G.M., Cumbo E.M., Luciani A., Gallina G., Mammina C., Pizzo G. (2009). *In vitro* evaluation of the antibacterial activity of cured dentin/enamel adhesive incorporating the antimicrobial agent MDPB. New Microbiol.

[61] Imazato S., Imai T., Russell R.R., Torii M., Ebisu S. (1998). Antibacterial activity of cured dental resin incorporating the antibacterial monomer MDPB and an adhesion-promoting monomer. J. Biomed. Mater. Res.

[62] Geurtsen W., Leyhausen G. (2001). Concise review biomaterials & bioengineering: chemical-biological interactions of the resin monomer triethyleneglycol-dimethacrylate (TEGDMA). J. Dent. Res.

[63] Thonemann B., Schmalz G., Hiller K.A., Schweikl H. (2002). Response of L929 mouse fibroblasts, primary and immortalized bovine dental papilla-derived cell lines to dental resin components. Dent. Mater.

[64] Ebi N., Imazato S., Noiri Y., Ebisu S. (2001). Inhibitory effects of resin composite containing bactericide-immobilized filler on plaque accumulation. Dent. Mater.

[65] Tanner J., Robinson C., Soderling E., Vallittu P.K. (2005). Early plaque formation on fibre-reinforced composites *in vivo*. Clin. Oral Investig.

[66] Lassila L.V., Garoushi S., Tanner J., Vallittu P.K., Soderling E. (2009). Adherence of *Streptococcus mutans* to fiber-reinforced filling composite and conventional restorative materials. Open Dent. J.

